# Quality of Life and Behavioural Self-Regulation Style in Different Groups of the Russian Population during the Second Wave of COVID-19

**DOI:** 10.1192/j.eurpsy.2025.1887

**Published:** 2025-08-26

**Authors:** V. I. Rozhdestvenskiy, V. V. Titova, I. A. Gorkovaya, Y. S. Aleksandrovich, D. O. Ivanov

**Affiliations:** 1 Department of Psychosomatics and Psychotherapy, Saint Petersburg State Pediatric Medical University, Saint Petersburg, Russian Federation

## Abstract

**Introduction:**

Quality of life (QoL) is a comprehensive concept encompassing an individual’s satisfaction with various aspects of life, including material, social, spiritual needs, intellectual and physical development, and safety. During the COVID-19 pandemic, the quality of life in Russia declined due to environmental and social disruptions. Behavioural self-regulation, which reflects an individual’s ability to manage internal and external conditions, plays a key role in adapting to challenging situations. Thus, exploring the relationship between QoL and self-regulation styles can provide insight into adaptive behaviours under pandemic conditions.

**Objectives:**

The study aimed to explore the interconnections between quality of life and self-regulation styles in humanities students and people living with HIV during the second wave of COVID-19.

**Methods:**

Data were collected from January to July 2021 via a Google form. Participants included 35 Russian university students in humanities and 59 HIV-positive patients. Self-regulation styles were measured using V.I. Morosanova’s “Style of Self-Regulation of Behaviour” questionnaire, and quality of life was assessed with the WHOQOL-BREF, adapted for Russian respondents.

**Results:**

In the group of students positive correlations of physical and psychological well-being with programming (r_s_ = 0.405, p < 0.05); self-perception — with programming (r_s_ = 0.522, p < 0.01), evaluation of results (r_s_ = 0.586, p < 0.01) and general level of self-regulation (r_s_ = 0.389, p < 0.05); microsocial support — with evaluation of results (r_s_ = 0.336, p < 0.05) were found. In the patient group, physical and psychological well-being were associated with outcome evaluation (r_s_ = 0.343, p < 0.01); self-image — with modelling (r_s_ = 0.605, p < 0.01), outcome evaluation (r_s_ = 0.467, p < 0. 01), flexibility (r_s_ = 0.444, p < 0.01) and overall level of self-regulation (r_s_ = 0.439, p < 0.01); microsocial support — with modelling (_s_ = 0.366, p < 0.01); social well-being — with modelling (r_s_ = 0.442, p < 0.01) and flexibility (r_s_ = 0.346, p < 0.01).

**Conclusions:**

The study found that self-perception was the most frequently correlated factor with self-regulatory behaviour in both students and HIV-positive group, indicating that satisfaction with life, sense of purpose, and emotional stability contribute to self-regulation even in challenging conditions. However, social well-being was a unique influencing factor for people living with HIV, highlighting a dependency on material and societal conditions that was less pronounced in student’s group. This suggests that HIV patients are more sensitive to social and environmental stability, whereas students rely more on internal self-regulatory mechanisms for adaptation.

**Disclosure of Interest:**

None Declared

